# Amyloid beta-correlated plasma metabolite dysregulation in Alzheimer's disease: an untargeted metabolism exploration using high-resolution mass spectrometry toward future clinical diagnosis

**DOI:** 10.3389/fnagi.2023.1189659

**Published:** 2023-06-29

**Authors:** Jingzhi Yang, Shuo Wu, Jun Yang, Qun Zhang, Xin Dong

**Affiliations:** ^1^Institute of Translational Medicine, Shanghai University, Shanghai, China; ^2^Neurology Department, Shanghai Baoshan Luodian Hospital, Shanghai, China; ^3^Department of Internal Medicine, Shanghai Baoshan Elderly Nursing Hospital, Shanghai, China; ^4^School of Medicine, Shanghai University, Shanghai, China; ^5^Suzhou Innovation Center of Shanghai University, Suzhou, Jiangsu, China

**Keywords:** Alzheimer's disease, neurodegenerative biomarkers, human plasma, high-resolution mass spectrometry, clinical diagnosis

## Abstract

**Introduction:**

Alzheimer's disease (AD) is a leading cause of dementia, and it has rapidly become an increasingly burdensome and fatal disease in society. Despite medical research advances, accurate recognition of AD remains challenging. Epidemiological evidence suggests that metabolic abnormalities are tied to higher AD risk.

**Methods:**

This study utilized case-control analyses with plasma samples and identified a panel of 27 metabolites using high-resolution mass spectrometry in both the Alzheimer's disease (AD) and cognitively normal (CN) groups. All identified variables were confirmed using MS/MS with detected fragmented ions and public metabolite databases. To understand the expression of amyloid beta proteins in plasma, ELISA assays were performed for both amyloid beta 42 (Aβ42) and amyloid beta 40 (Aβ40).

**Results:**

The levels of plasma metabolites PAGln and L-arginine were found to significantly fluctuate in the peripheral blood of AD patients. In addition, ELISA results showed a significant increase in amyloid beta 42 (Aβ42) in AD patients compared to those who were cognitively normal (CN), while amyloid beta 40 (Aβ40) did not show any significant changes between the groups. Furthermore, positive correlations were observed between Aβ42/Aβ40 and PAGln or L-arginine, suggesting that both metabolites could play a role in the pathology of amyloid beta proteins. Binary regression analysis with these two metabolites resulted in an optimal model of the ROC (AUC = 0.95, *p* < 0.001) to effectively discriminate between AD and CN.

**Discussion:**

This study highlights the potential of advanced high-resolution mass spectrometry (HRMS) technology for novel plasma metabolite discovery with high stability and sensitivity, thus paving the way for future clinical studies. The results of this study suggest that the combination of PAGln and L-arginine holds significant potential for improving the diagnosis of Alzheimer's disease (AD) in clinical settings. Overall, these findings have important implications for advancing our understanding of AD and developing effective approaches for its future clinical diagnosis.

## 1. Introduction

Alzheimer's disease (AD) is a form of neurological dementia that is progressive and irreversible; it has a significant negative impact on people's lives, society, and the economy (2021). Numbers of biochemical processes are affected in AD pathologies, which include the breakdown of amyloid precursor proteins, the phosphorylation of tau proteins, oxidative stress, poor energy, mitochondrial dysfunction, inflammation, membrane lipid dysregulation, or disruption of neurotransmitter pathways (de la Monte and Tong, 2014; Procaccini et al., 2016). It is increasingly clear that many neurodegenerative diseases have a pre-symptomatic phase, during which pathological changes accumulate prior to the onset of symptoms (Golde, 2022). Thereof, early diagnosis and therapy during the progression of AD are critical and a rapid pace of development should be adopted (Cummings et al., 2022). Nevertheless, the challenge of obtaining a prompt and accurate diagnosis has hindered the development of therapies for Alzheimer's disease (AD). As reported in a previous study, AD clinical trials are characterized by up to 80% screen failure rates (Aisen et al., 2016). Biomarkers enable the identification of the onset, profile, and severity of neurodegeneration-related brain alterations in particular patients who are in need of diagnosis, prognosis, and usage in clinical trials—as both inclusion and outcome measures—as the fieldwork to treat patients sooner and earlier (Bendlin and Zetterberg, 2022). The National Institute on Aging and Alzheimer's Association (NIA-AA) has proposed a research framework for using A/T/N biomarkers of β amyloid, tau, and neurodegeneration biomarkers to define Alzheimer's disease. These A/T/N biomarkers shall also serve as continuous measures to reflect different cognitive stages (Jack et al., 2018).

Currently, the availability of amyloid beta (A) PET and cerebrospinal fluid (CSF) biomarker tests for amyloid beta peptides, tau, and other neuroproteins (A/T/N classifiers) enables their use to diagnose brain amyloid pathology. However, there is still an unmet need for an accessible, radiation-free, minimally invasive, economical, quick, and analytically validated diagnostic approach to simplify clinical trial enrollment (Jack et al., 2016). Besides, the biochemical and physiological changes in the brain that characterize the illness beyond amyloid and tau deposition are still poorly understood, even though AD is currently characterized based on amyloid-plaque and tau neurofibrillary tangle deposition inside the neocortex (Jack et al., 2018).

Metabolome analysis has emerged as a novel strategy for the development of disease biomarkers in diagnosis, as well as for monitoring the progression of the disease with its underlying pathophysiology (Trivedi et al., 2017). The metabolome is a collection of small molecules that is produced by metabolic processes, arranged in biochemical pathways. It is impacted by various internal and external variables, including genetics (Holmes et al., 2008). It has been indicated that metabolomics appears to be of uttermost relevance in AD as several metabolic changes, such as higher insulin and insulin resistance levels, are associated with an increased risk of AD (Schrijvers et al., 2010). Thus, metabolites are now crucial diagnostic indicators of dementia before memory loss, defining its presence or absence.

As the plasma metabolome interacts and exchanges molecules with every organ and tissue, including the brain, it reflects various physiological and pathological changes. This makes it a promising avenue for identifying biomarkers for a range of disorders. Furthermore, interorgan communication is an important and conserved mechanism that maintains body homeostasis. Dysregulation of the systemic homeostatic system would result in metabolic and neurological disorders (Vogt and Bruning, 2013; Deleidi et al., 2015). Moreover, plasma is a bodily fluid that is simple to obtain and causes minimal discomfort to patients, which allows for the collection from large cohorts and repeated sampling (Lawton, 2008). Therefore, it is worth studying the systemic changes in blood metabolite levels associated with AD.

Analytical techniques have significantly improved, with high-resolution mass spectrometry (HRMS) instruments being readily available for determining the majority of chemical compounds (Niedzwiecki et al., 2020). Apart from determining the accurate properties of these metabolites, collective quantification is of great importance for metabolism study (Koek et al., 2011). The most popular mass spectrometers for UHPLC-HRMS are Orbitrap (OT) or TOF-based systems, as they enable the best MS data acquisition. However, according to instrument investigation, the resolution of a UHPLC-coupled OT instrument has been sacrificed in favor of achieving greater separation with higher acquisition rates (Kaufmann, 2018; de Souza et al., 2021). Furthermore, in line with our previous research on the discovery of AD urine metabolites, the OT systems for molecule detection displayed impressive stability performance (Zhang et al., 2022). Moreover, recent research that employs powerful bioinformatic techniques and high-throughput measurements of hundreds of metabolites has thoroughly documented the molecular alterations and disease-related pathways (Chandler et al., 2016; Sales et al., 2017; Uppal et al., 2017; Zhuang et al., 2021).

We performed a metabolomic analysis of the plasma of patients with AD and CN using high-resolution mass spectrometry (HRMS) from these viewpoints. Our current investigation supports the application of metabolomics analysis as a discrimination test between AD and CN, which may provide new insight for future clinical diagnosis.

## 2. Methods

### 2.1. Participant ascertainment and ethics approval

During the visits to the Shanghai Baoshan Senior Care Home, Baoshan District, No. 5425 Gonghe New Road, individuals between 60 and 80 years of age were recruited as participants, and a wide range of biospecimens and health indicators were collected. Standard cognitive screening, which includes medical history assessment, cognitive examination, and blood sampling, was conducted for all patients with Alzheimer's disease (AD). Regular biomedical indicators were obtained for cognitively normal (CN) individuals. The ethics committee of Shanghai Baoshan Luodian Hospital approved this study prior to the acquisition of clinical and genetic participants' data (Approval number: LDYY-KY-2020-04). The study was performed in accordance with the ethical standards laid down in the 1964 Declaration of Helsinki and its later amendments.

### 2.2. ADAS-Cog assessment and participants' grouping

All study participants were fully informed of this research work, and written informed consent was obtained from all participants. The Alzheimer's Disease Assessment Scale—Cognitive (ADAS-Cog) Subscale test is a widely used cognitive test in research studies and clinical trials (Kueper et al., 2018; Zhang et al., 2022) and, therefore, was administered to evaluate the participants' recognition ability before the wet-lab experiments. Scores on the ADAS-Cog test range from 0 to 75, with a score above 18 indicating recognition impairment and leading to enrollment in the AD group. Conversely, a score below 18 indicates normal recognition ability and leads to enrollment in the CN group.

### 2.3. Plasma samples collection

Briefly, blood was collected in the morning, following an overnight fast of at least 8 h. EDTA blood tubes were used for plasma collection, which were then centrifuged at a speed of 3,000 g for 15 min at room temperature. The resulting supernatant was transferred and aliquoted into polypropylene tubes of 0.5 mL and stored at −80°C until further use. Quality control (QC) plasma was prepared by pooling an equal amount of individual plasma samples and was utilized to assess downstream sample preparation and MS measurements' stability.

### 2.4. Plasma protein biomarkers quantification

Plasma contents of total tau, APOE, amyloid beta-peptide 1-40, and amyloid beta-peptide 1-42 were measured using commercial ELISA kits according to the manufacturer's instructions (Total Tau, KHB0041, Invitrogen; APOE, ELH-ApoE4-1, RayBiotech; Amyloid beta 1-40, RE59781, IBL, and Amyloid beta 1-42, KHB3544, Thermo Fisher Scientific). Briefly, standard assays for detecting radioimmunoprecipitation assay-soluble samples were applied to the ELISA plates. After washing, a biotin-conjugated detection antibody was applied. Then, the positive reaction was enhanced with streptavidin–horseradish peroxidase and colored by 3,3′,5,5′-tetramethylbenzidine. The absorbance at 450 nm was applied, and the concentrations of four different proteins were calculated from the standard curves. All measurements were carried out in one round of experiments, and the results were read on a microplate photometer (Multiskan^TM^ FC, Thermo Fisher).

### 2.5. HRMS on untargeted metabolomics

The aliquoted frozen plasma sample was thawed and centrifuged at 14,000 g for 5 min. A measure of 300 microliters of methanol was added to 100 μL aliquot of plasma samples, vortexed for 5 min, and centrifuged at 14,000 g for another 5 min. The supernatant was transferred into a plastic tube, evaporated to dryness under a stream of nitrogen at 40°C, and reconstituted in 100 μL of acetonitrile, which contains 5 μg/mL 2-Chloro-L-phenylalanine (Sigma-Aldrich). A 5.0-μL aliquot of the reconstituted solution was injected into the UPLC MS system for online data acquisition. The same sample preparation steps were applied for QC samples. The plasma metabolites were separated on a Waters HPLC Column (XSelect HSS T3, 2.1 X 100 mm, 2.5 μm, MA, USA) that equilibrated at 37°C. The mobile phase consisted of 0.1% formic acid in water (A) as the aqueous phase and 0.1% formic acid in acetonitrile as the organic phase. The gradient elution (min, B) was set as 20 min: 0.0–2.0, 5%; 2.0–6.0, 50%; 6.0–15.0, 95%; 15.0–18.0, 95%; and 18.0–20.0, 5%. The eluent flow rate was set to 0.3 mL/min.

Sample extracts were analyzed using UPLC interfaced with the high-resolution MS system of Orbitrap (Dionex Ultimate 3000, Q-Exactive Plus, Thermo Scientific) in the positive electrospray ionization (ESI+) mode. The mass range was set to m/z 65–975. For the MS scan, the MS resolution was set to 35,000 with the automatic gain control (AGC) target set to 1 × 10^6^ and the maximum ions injection time was set to 100 ms. For the MSMS scan, the MS resolution was set to 17,500 with the automatic gain control (AGC) target set to 1 × 10^5^, and the maximum ion injection time was set to 50 ms. The stepped normalized collision energy (NCE) consisted of 20%, 25%, and 30% for ion fragmentation, and the isolation window was narrowed to 1.0 m/z for improving the MS feature identification. MS injection order followed the previous batch sequence setting (Zhang et al., 2022). In brief, QC samples were placed at the beginning, in between the samples, and at the end of the whole batch to examine the stability of the MS method.

### 2.6. Database search, data cleaning, and evaluation

The MS data underwent processing using Thermo Compound Discover 3.1 (Thermo Scientific, USA). An “Untargeted metabolomics workflow” was used to extract MS features and identify the nature of the compounds. In brief, MS raw files including QCs, ADs, and CNs were introduced into the data study. A specific sample type was selected for each of the individual raw data. The custom “Workflow Tree” was optimized according to the HRMS settings. Databases of mzCloud and mzVault were selected for compound identification. Then, the analysis was submitted to the job queue and resulted in a list with compound features, MS intensities, retention time (RT), MSMS spectrum, and so on. According to the database search results, MS features with more than 20% missing values were removed as these signals' quality was deemed uncertain for further validation and quantification. Also, those calculated MS intensities of lower than 10,000 were not included as their plasma levels were too low to be quantified with this method. To evaluate the stability of this untargeted approach, the intensities of selected metabolites and the internal standard (2-Chloro-L-phenylalanine) in all QC samples were analyzed.

### 2.7. Metabolome-wide association study

Metabolome-wide association study (MWAS) was conducted with Simca-P 14.1 software (Umetrics, Umea, Sweden) (Wheelock and Wheelock, 2013). Data of normalized LC/MS peak areas were imported for multivariate analysis. Principal component analysis (PCA), partial least-squares discriminant analysis (PLS-DA), and orthogonal partial least-squares discriminant analysis (OPLS-DA) were performed separately for model development. The quality of the model was tested by cross-validation and permutation (Szymańska et al., 2012) and evaluated by the values of R^2^X, R^2^Y, and Q^2^. By default, the model was run through seven rounds of cross-validation to establish the optimal number of principal components to minimize overfitting and the fact that both Q^2^ and R^2^ were near to 1, which shows that the model is excellent (Liang et al., 2011).

### 2.8. Metabolic pathway analysis

A metabolic pathway may be conceived of as a group of metabolites that arise from various regions of the metabolome and cooperate to control the processes of AD. Moreover, we looked at regulatory signatures related to the AD disease process using network extraction approaches. Thus, the Mummichog analysis was carried out using MetaboAnalyst (version 5.0). To identify m/z characteristics with a statistical significance of *p* < 0.05, Student's *t*-test analysis was performed. Features of m/z with calculated significance were then matched to the metabolic models of Kyoto Encyclopedia of Genes and Genomes (KEGG). Following validation of the m/z features that were mapped onto the metabolite networks, statistically significant values were reported. We took advantage of that technique to present further data on potential metabolic variations between AD and CN.

### 2.9. Metabolites MS/MS validation and semi-quantification

Metabolite annotation and identification were performed using both spectra of MS and MS/MS, which were further validated with the HMDB (https://hmdb.ca/) and PubChem (https://pubchem.ncbi.nlm.nih.gov/) databases. The MS and MS/MS fragments were all examined in each of the individual MS spectra. Discriminatory features that were associated with the significantly enriched pathways and a *p* < 0.05 were selected for semi-quantitation analysis. The semi-quantification was performed by calculating the relative responses (Rel. Res) of each metabolite, and the equation is listed as follows:


$$\begin{array}{l} \;Relative\;response\left(Rel\text{. }Res\text{.}\right)\\ =Intenstiy\frac{validated\;metabolite}{Internal\;standard\;\left(2-Chloro-L-phenylalanine\right)}\; \end{array}$$

Further, the calculated relative responses were used to investigate the statistical differences between the AD and CN groups.

### 2.10. Statistical analysis

Continuous variables were compared using Student's *t*-test or the Mann–Whitney *U*-test. The area under the receiver operating characteristic curve [ROC (AUC)] was calculated to perform the discrimination power of the potential biomarkers for AD. Differential expression with metabolites and proteins between the AD and CN groups was demonstrated as boxplots. The correlation analysis was conducted between AD metabolites and protein biomarkers. Analyses were performed using GraphPad Prism (version 9.0.0, San Diego, USA). A *p* < 0.05 was considered statistically significant.

## 3. Results

### 3.1. Demographics characteristics of participants

Based on the results of the Alzheimer's Disease Assessment Scale—Cognitive (ADAS-Cog) Subscale, 29 participants scored between 53 and 75 and were classified as belonging to the Alzheimer's disease (AD) group, with an average score of 72.65 ± 5.56. Additionally, 29 participants scored between 0 and 16.5 and were included in the cognitively normal (CN) group, with an average score of 6.48 ± 6.45. Among the study participants, 63.3% of females were in the AD group, while 58.6% were in the CN group. Although the average education year was longer in the CN group (6y ± 3) than in the AD group (4y ± 2), there was no significant difference between the two groups. The average ages of the AD and CN groups were 69.0 years ± 3.60 and 72.1 years ± 7.1, respectively, with no significant difference in the age distribution between the two groups (Table 1).

**Table 1:** Demographic characteristics of the AD and CN groups.

**Characteristics**	**AD group**	**CN group**
*n*	29	29
Gender, female/male (%)	63.3	58.6
Age	69.0 ± 3.60	72.1 ± 7.1
Education year (y)	4 ± 2	6 ± 3
ADAS-Cog score (Score range)	72.65 ± 5.56 (53–75)	6.48 ± 6.45 (0–16.5)

Results are expressed as mean ± standard deviation.

### 3.2. AD protein biomarkers measurements

The biomarkers for AD protein, such as Amyloid beta 1-42 (Aβ42), Amyloid beta 1-40 (Aβ40), t-tau, and APOE, were measured in both the AD and CN groups, and the Aβ42/Aβ40 ratio was calculated. The plasma concentration of Aβ42 was noted to increase while the plasma concentrations of t-tau and APOE were observed to decrease significantly (*p* < 0.0001) in individuals with AD as compared to those without (CN). However, no significant difference was found in Aβ40 levels between the AD and CN groups. These observations are tabulated in Table 2 and are depicted as comparative boxplots in Supplementary Figure 1.

**Table 2:** Plasma protein feature measurements by ELISA.

**Protein features (unit)**	**AD group**	**CN group**	***p*-value**
Aβ40 (pg/mL)	333.908 ± 94.157	303.067± 114.857	0.142
Aβ42 (pg/mL)	9.422 ± 11.433	3.725 ± 2.722	0.001
TAU (pg/mL)	1,199.255 ± 959.953	2,217.143 ± 975.046	0.000
APOE (ng/mL)	32,617.841 ± 9,475.138	66,157.364 ± 108,855.517	0.002
Aβ42/Aβ40	0.027 ± 0.029	0.013 ± 0.010	0.001

Results are expressed as mean ± standard deviation; a *p* < 0.05 indicates statistical significance.

### 3.3. MWAS results

**Figure 1:**
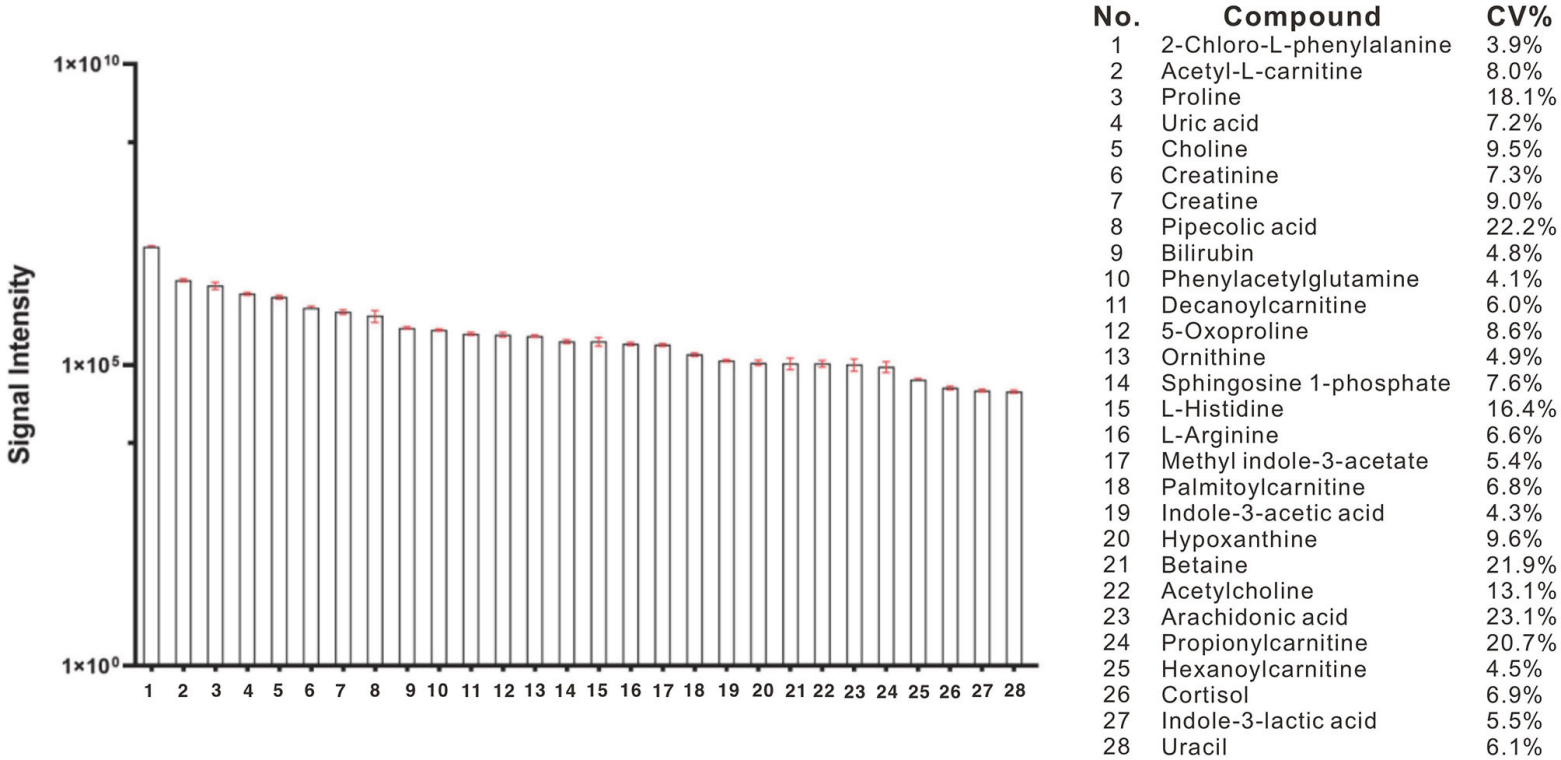
Bar column of average intensities and standard deviations of 28 compounds, including 27 metabolites and an internal standard of 2-Chloro-L-phenylalanine in all QC samples across the entire MS-analytical batches.

Using a high-resolution MS platform for the purpose of metabolome profiling, 612 distinct m/z characteristics with projected formulas or chemical names were acquired, where only those characteristics with an average batch determination rate of 80% or above were considered for further study (data are shown in Supplementary Table 1). Subsequently, a selection of the relative intensities was made, where those with CV% values <30% were chosen as criteria for further investigation, ultimately narrowing the list of chemicals down to 42. The databases of HMDB and PubChem were then utilized to validate the compound resources and MSMS fragmentation. This process resulted in the formation of a final endogenous compound list of 27 metabolites for the investigation of metabolite expression in both AD and CN groups (Table 3). The CV% of QC results was analyzed to ensure a convincing result in downstream analysis. As a result, the MS areas of nine QC samples were calculated for 27 metabolites and one internal standard, where the maximum value of the CV% is 23.1% for arachidonic acid and the minimum value of the CV% is 3.9% for 2-Chloro-L-phenylalanine. On average, the CV% value of the 28 compounds is 9.7%, indicating the MS measurement has good stability (refer to Table 4). Additionally, a plot of the average signal intensities and their standard deviations of 28 compounds are depicted in Figure 1.

**Table 3:** Mass spectrometric characteristics of plasma metabolites and internal standard.

**No**.	**Compound name**	**HMDB ID**	**KEGG ID**	**PubChem CID**	**Formula**	**MW**	**RT (min)**	**[M+H]^+^**	**Identified MSMS fragment ions**
1	Phenylacetylglutamine	HMDB06344	C04148	92258	C13 H16 N2 O4	264.11083	5.554	265.1183	223/115/101
2	L-Arginine	HMDB0000517	C00062	6322	C6 H14 N4 O2	174.11156	0.764	175.1191	175/116/130
3	Propionylcarnitine	HMDB0000824	C03017	107738	C10 H19 N O4	217.13121	0.977	218.1387	159/144
4	Creatine	HMDB0000064	C00300	586	C4 H9 N3 O2	131.06952	0.834	132.0769	132/114/100
5	Creatinine	HMDB0000562	C00791	588	C4 H7 N3 O	113.05909	0.821	114.0664	114/86/72
6	Indole-3-acetic acid	HMDB0000197	C00954	802	C10 H9 N O2	175.06323	7.021	176.0706	103/102/99
7	Pipecolic acid	HMDB0000070	C00408	849	C6 H11 N O2	129.07892	0.696	130.0863	130/110/84
8	Arachidonic acid	HMDB0001043	C00219	444899	C20 H32 O2	304.23991	15.685	305.2473	93/117/105
9	Choline	HMDB0000097	C00114	305	C5 H13 N O	103.10007	0.791	104.1073	60/58
10	Indole-3-lactic acid	HMDB0000671	C02043	92904	C11 H11 N O3	205.07378	6.52	206.0812	188/160/130
11	Proline	HMDB0000162	C00148	145742	C5 H9 N O2	115.06354	0.842	116.0706	70/116/68
12	Acetylcholine	HMDB0000895	C01996	187	C7 H15 N O2	145.11017	0.832	146.1150	87/60/43
13	Betaine	HMDB0000043	C00719	247	C5 H11 N O2	117.0791	5.575	118.0863	56/59/119
14	Bilirubin	HMDB0000054	C00486	5280352	C33 H36 N4 O6	584.26342	7.658	585.2705	285/539/253/286
15	Methyl indole-3-acetate	HMDB0029738	NA^a^	74706	C11 H11 N O2	189.07887	7.525	190.0861	130/172/101
16	Cortisol	HMDB0000063	C00735	5754	C21 H30 O5	362.20892	7.307	363.2160	327/309/121
17	L-Histidine	HMDB0000177	C00135	6274	C6 H9 N3 O2	155.06942	0.715	156.0767	125/84/79
18	Hypoxanthine	HMDB0000157	C00262	135398638	C5 H4 N4 O	136.03844	1.241	137.0457	137/119/95/81
19	Hexanoylcarnitine	HMDB0000756	NA^a^	6426853	C13 H25 N O4	259.17816	6.122	260.1856	85/201/99
20	Uric acid	HMDB0000289	C00366	1175	C5 H4 N4 O3	168.0283	1.239	169.0357	152/141
21	Ornithine	HMDB0000214	C00077	6262	C5 H12 N2 O2	132.08996	0.696	133.0983	125/79/84
22	Uracil	HMDB0000300	C00106	1174	C4 H4 N2 O2	112.02747	1.292	113.0348	96/70
23	Acetyl-L-carnitine	HMDB0000201	C02571	7045767	C9 H17 N O4	203.11572	0.891	204.1230	85/145
24	Decanoylcarnitine	HMDB0000651	C03299	10245190	C17 H33 N O4	315.24072	7.796	316.2482	85/257/155
25	Palmitoylcarnitine	HMDB0000222	C02990	461	C23 H45 N O4	399.33459	10.967	400.3419	85/341/239
26	5-Oxoproline	HMDB0000267	C01879	7405	C5 H7 N O3	129.04261	1.274	130.0500	84/85/131
27	Sphingosine 1-phosphate	HMDB0000277	C06124	5283560	C18 H38 N O5 P	379.24852	9.61	380.2557	346/223/101
28^b^	2-Chloro-L-phenylalanine	NA^a^	NA^a^	2761491	C9 H10 Cl N O2	199.04012	4.764	200.0472	154/183/118/165

^a^NA, not available.

^b^Internal standard.

**Table 4:** Mass spectrometric signal intensities of 28 compounds in quality control (QC) samples across the analytical batch.

**No**.	**Compound Name**	**HRMS signal intensity**									**Calculated value**		
		**QC 1**	**QC 2**	**QC 3**	**QC 4**	**QC 5**	**QC 6**	**QC 7**	**QC 8**	**QC 9**	**Average**	**SD**	**CV%**
1	2-Chloro-L-phenylalanine	9529581	9088904	9485429	8742235	9469332	9395555	9548525	8850665	8704423	9,201,628	355,641	3.9%
2	Phenylacetylglutamine	382822	383624	392065	384992	387027	384947	373850	393136	341656	380,458	15,589	4.1%
3	Indole-3-acetic acid	124032	119284	121262	120338	114885	122387	107521	115021	118044	118,086	5,021	4.3%
4	Hexanoylcarnitine	58657	60892	58681	54927	57700	55457	54468	52743	56352	56,653	2,543	4.5%
5	Bilirubin	406307	399800	418485	422007	452406	407273	422593	383009	399875	412,417	19,611	4.8%
6	Ornithine	298137	292367	312863	298005	286066	294694	333486	295200	315194	302,890	14,805	4.9%
7	Methyl indole-3-acetate	226667	226120	228808	211032	224630	215322	215505	197252	199534	216,097	11,740	5.4%
8	Indole-3-lactic acid	39725	40016	38566	37977	34284	39277	35479	37280	35526	37,570	2,066	5.5%
9	Decanoylcarnitine	332006	330514	312064	327887	363043	342753	352238	303945	309703	330,461	19,901	6.0%
10	Uracil	34652	38553	34294	38542	33435	37115	33404	38183	37381	36,173	2,199	6.1%
11	L-Arginine	227217	200801	213641	226435	234702	207762	248974	233852	227311	224,522	14,871	6.6%
12	Palmitoylcarnitine	162114	139816	153831	152894	151122	149560	151737	158446	128209	149,748	10,146	6.8%
13	Cortisol	46274	42796	42073	37517	40200	45271	41367	41043	38296	41,649	2,891	6.9%
14	Uric acid	1467068	1499030	1419389	1601035	1732179	1657507	1486082	1505374	1406219	1,530,431	110,221	7.2%
15	Creatinine	866914	768723	865649	884379	862536	876028	894620	948036	1007037	885,991	64,959	7.3%
16	Sphingosine 1-phosphate	262005	266871	262232	240920	252065	242596	248322	230150	207334	245,833	18,644	7.6%
17	Acetyl-L-carnitine	2827805	2551314	2853465	2304034	2525169	2693666	2380845	2599126	2319824	2,561,694	204,268	8.0%
18	5-Oxoproline	293296	320502	273675	304564	321100	360339	338945	350682	314297	319,711	27,519	8.6%
19	Creatine	624589	755177	749724	796530	718671	748896	850668	752585	845281	760,236	6,8172	9.0%
20	Choline	1237398	1109407	1409400	1374631	1411754	1559393	1309037	1272301	1384232	1,340,839	127,715	9.5%
21	Hypoxanthine	108960	94697	93750	104105	122321	122007	110056	108137	117448	109,053	10,494	9.6%
22	Acetylcholine	111943	106859	124111	104598	88129	86441	95092	109266	124226	105,629	13,823	13.1%
23	L-Histidine	183886	196229	236228	288127	257609	260952	214771	274265	297919	245,554	40,360	16.4%
24	Proline	2299600	1450474	2030671	1514785	2035300	2526576	2160492	2444573	2252567	2,079,449	376,999	18.1%
25	Propionylcarnitine	113886	123703	94571	103424	105471	86245	65572	76310	75260	93,827	19,463	20.7%
26	Betaine	93875	137794	131380	114206	88957	114522	79435	128731	74935	107,093	23,476	21.9%
27	Pipecolic acid	274335	664566	749064	714482	704826	731427	705162	669263	700309	657,048	145,963	22.2%
28	Arachidonic acid	112458	120743	139285	116542	97998	101572	66210	93987	70134	102,103	23,556	23.1%

### 3.4. Discrimination model evaluation

**Figure 2:**
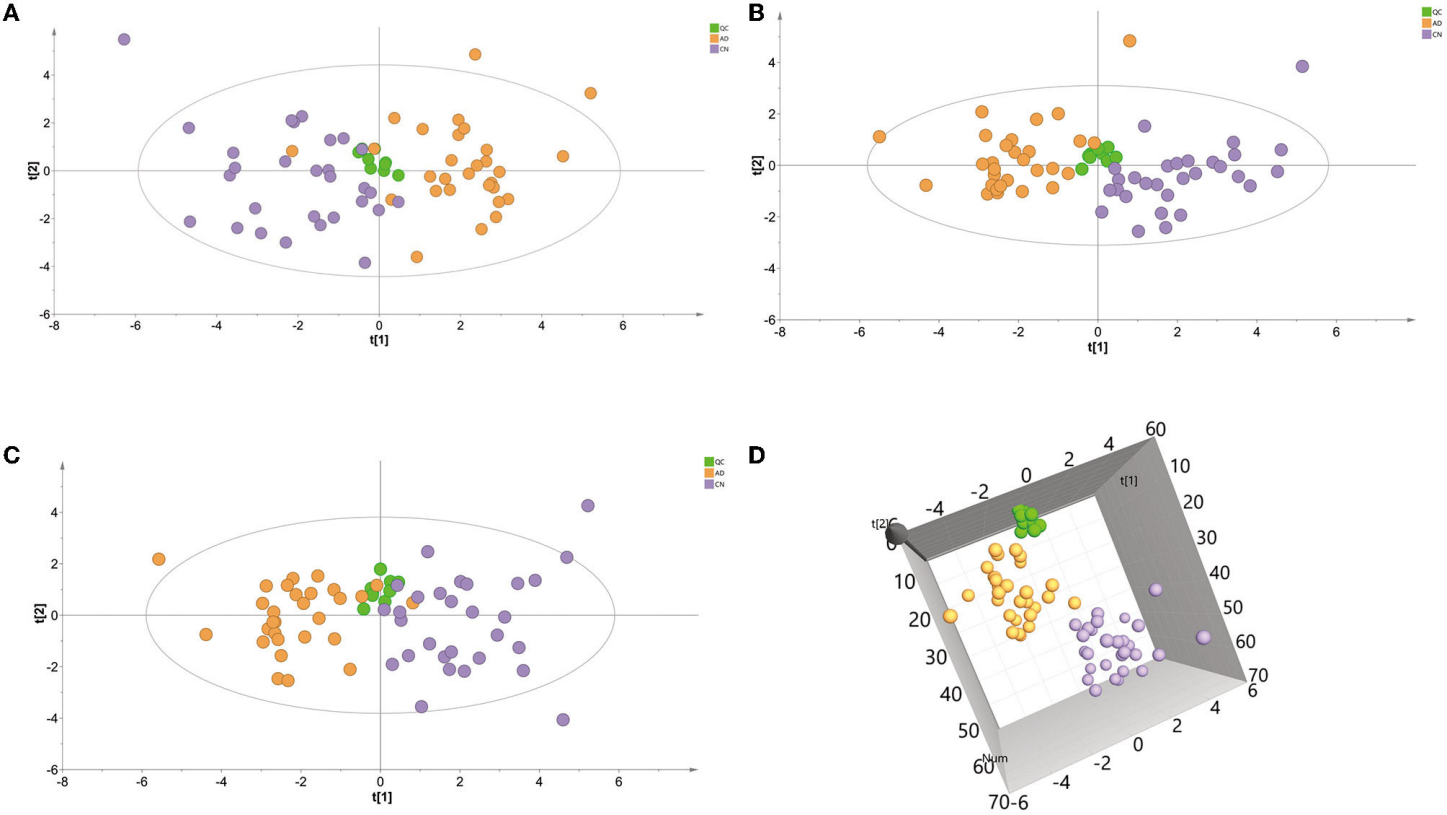
Multivariate data analysis of plasma samples in the ESI-positive mode. **(A)** Principal components analysis (PCA) score plot of the QC, AD, and CN groups. **(B)** Partial least-squares discriminant analysis (PLS-DA) score plot of the QC, AD, and CN groups. **(C)** Orthogonal partial least-squares discriminant analysis (OPLS-DA) score plot of the QC, AD, and CN groups. **(D)** A three-dimensional plot of the OPLS-DA model.

A principal component analysis (PCA) model was conducted with identified metabolites. The PCA (R^2^X = 0.331 Q^2^ = 0.081) scores plot showed an approximate separation between the AD and CN groups (Figure 2A), indicating a tendency of inter-group clustering. Additionally, the partial least-squares discriminant analysis (PLS-DA) model (R^2^X = 0.289, R^2^Y = 0.477, Q^2^ = 0.342) and orthogonal partial least-squares discriminant analysis (OPLS-DA) model (R^2^X = 0.297, R^2^Y = 0.456, Q^2^ = 0.35) were performed to compare the AD and CN groups. The corresponding score plots depicted in Figures 2B, C revealed a noticeable disjunction between the two groups. Moreover, a three-dimensional (3D) plot of the OPLS-DA model (Figure 2D) displayed a distinct separation between the AD and CN groups. These outcomes indicate plasma metabolic variations in AD patients.

### 3.5. Pathway analysis

**Figure 3:**
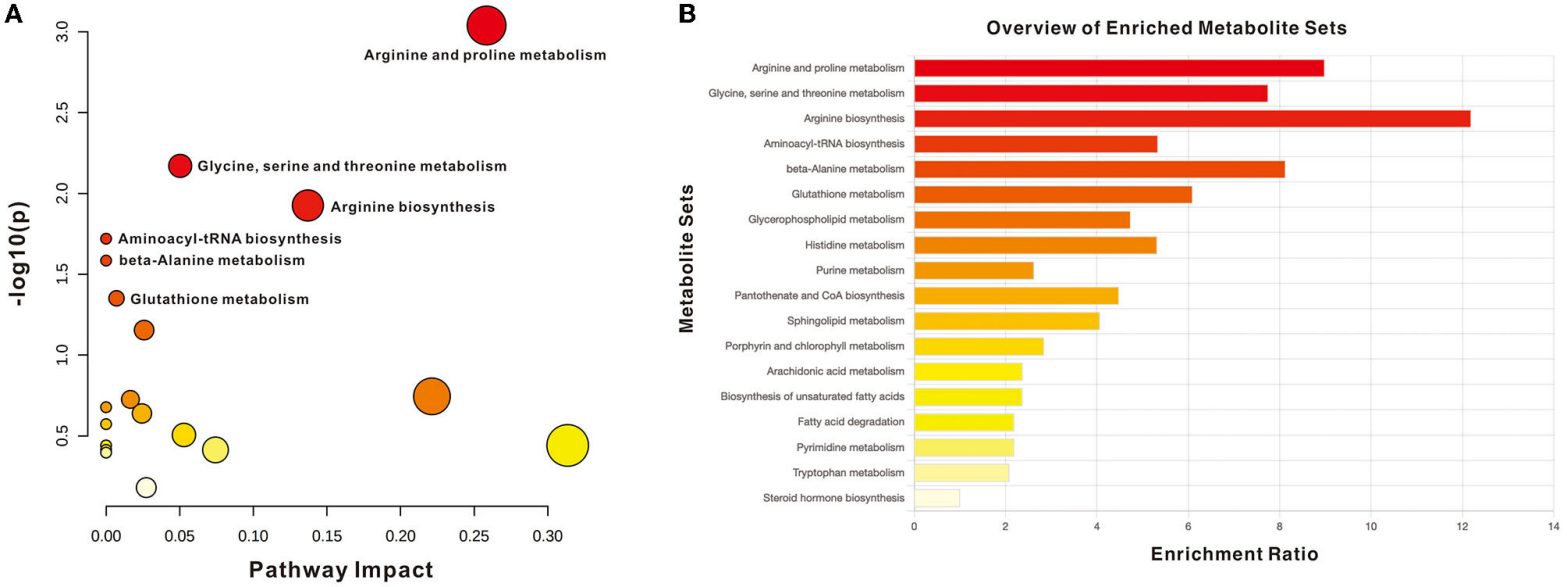
Summary of pathway analysis with MetaboAnalyst. **(A)** Pathway impact with significance (*p* < 0.05): arginine and proline metabolism, glycine, serine, and threonine metabolism, arginine biosynthesis, Aminoacyle-tRNA biosynthesis, and beta-alanine metabolism. **(B)** Metabolite sets enrichment overview.

The impact pathway was analyzed using MetaboAnalyst 5.0 (http://www.metaboanalyst.ca/). The results indicated that 27 endogenous metabolites were of close relevance to five biological pathways presenting statistical significance, which included the following: (1) arginine and proline metabolism, (2) glycine, serine, and threonine metabolism, (3) arginine biosynthesis, (4) aminoacyle-tRNA biosynthesis, and (5) beta-alanine metabolism. The *p*-values obtained for these metabolites were as follows: 0.0009, 0.0068, 0.0119, 0.0191, and 0.0260, respectively. The KEGG pathway produced a bubble plot, which is shown in Figure 3A, while the metabolic features' enrichment result is displayed in Figure 3B. All pathways and their related metabolites are summarized in Table 5.

**Table 5:** KEGG-enriched molecular pathway with identified metabolites.

**No**.	**Pathway name**	**Match status**	**Matched metabolites**	***p*-value**	**Impact**
1	Arginine and proline metabolism	4/38	L-Arginine, creatine, L-proline, and L-ornithine	0.0009	0.25841
2	Glycine, serine, and threonine metabolism	3/33	Choline, betaine, and creatine	0.0068	0.05034
3	Arginine biosynthesis	2/14	L-Arginine and L-ornithine	0.0119	0.13705
4	Aminoacyl-tRNA biosynthesis	3/48	L-Histidine, L-arginine, and L-proline	0.0191	0.00000
5	beta-Alanine metabolism	2/21	Uracil and L-histidine	0.0260	0.00000
6	Glutathione metabolism	2/28	5-Oxoproline and L-ornithine	0.0445	0.00709
7	Glycerophospholipid metabolism	2/36	Choline and acetylcholine	0.0700	0.02582
8	Histidine metabolism	1/16	L-histidine	0.1799	0.22131
9	Purine metabolism	2/65	Hypoxanthine and urate	0.1878	0.01651
10	Pantothenate and CoA biosynthesis	1/19	Uracil	0.2100	0.00000
11	Sphingolipid metabolism	1/21	Sphingosine 1-phosphate	0.2295	0.02434
12	Lysine degradation	1/25	L-pipecolic acid	0.2671	0.00000
13	Porphyrin and chlorophyll metabolism	1/30	Bilirubin	0.3117	0.05288
14	Biosynthesis of unsaturated fatty acids	1/36	Arachidonate	0.3618	0.00000
15	Arachidonic acid metabolism	1/36	Arachidonate	0.3618	0.3135
16	Fatty acid degradation	1/39	L-Palmitoylcarnitine	0.3856	0.00000
17	Pyrimidine metabolism	1/39	Uracil	0.3856	0.0743
18	Tryptophan metabolism	1/41	Indole-3-acetate	0.4009	0.00000
19	Steroid hormone biosynthesis	1/85	Cortisol	0.6597	0.02729

### 3.6. Potential plasma metabolic biomarkers of the AD and CN groups

**Figure 4:**
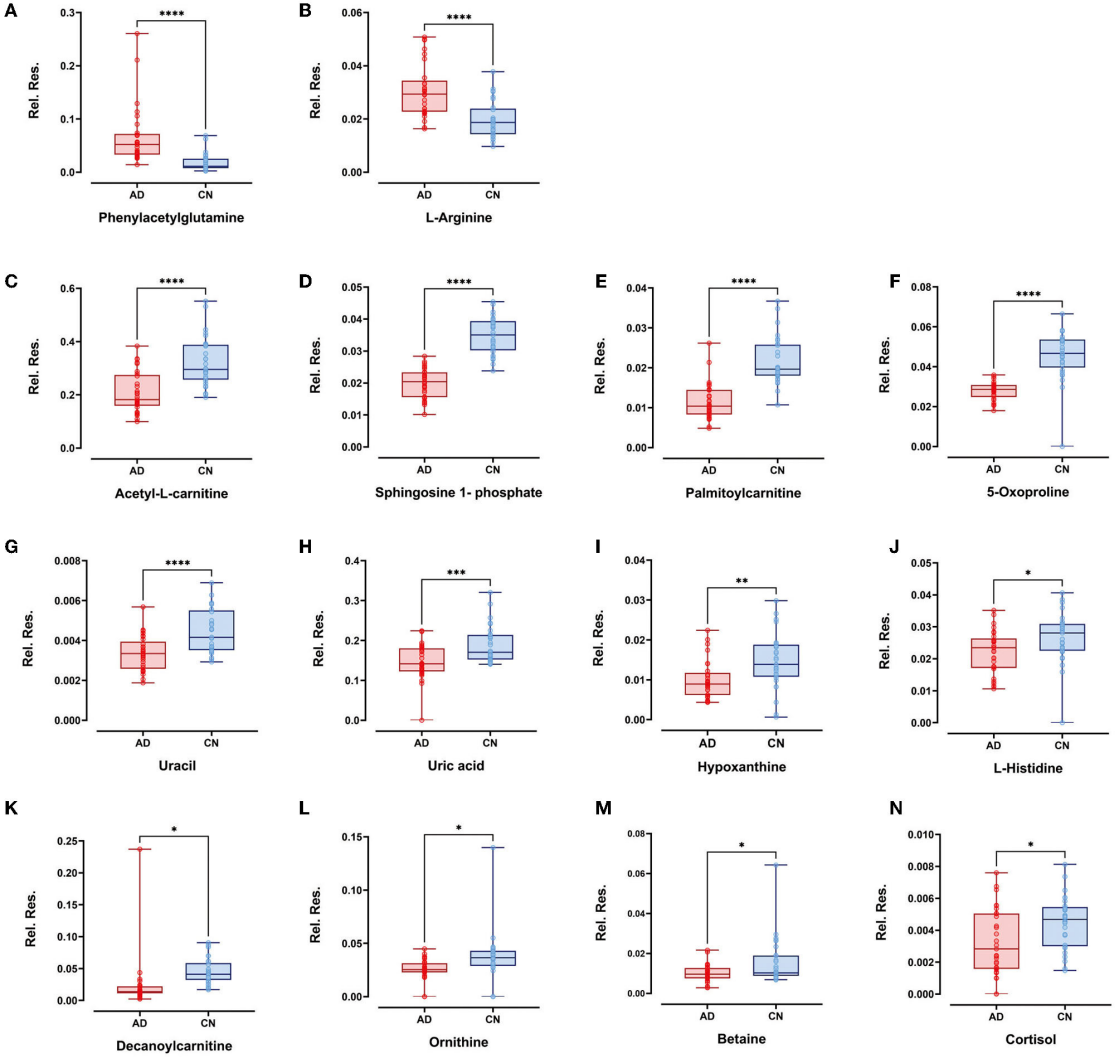
Boxplots showed upregulated plasma metabolites of **(A)** phenylacetylglutamine and **(B)** L-arginine (*p* < 0.0001). Boxplots showed downregulated plasma metabolites of **(C)** acetyl-L-carnitine, **(D)** sphingosine-1-phosphate, **(E)** palmitoylcarnitine, **(F)** 5-oxoproline, **(G)** uracil (*p* < 0.0001), **(H)** uric acid (*p* < 0.001); **(I)** hypoxanthine (*p* < 0.01); **(J)** L-histidine, **(K)** decanoylcarnitine, **(L)** ornithine, **(M)** betaine, and **(N)** cortisol (*p* < 0.05). Plots show data points from the minimum value to the maximum value for each group. Light pink dots represent AD patients, sky blue dots represent CN individuals, and dots were vertically aligned on the boxes for each group.

Within the selected 27 endogenous metabolites, 14 compounds exhibited different regulation trends between the AD and CN groups. Specifically, phenylacetylglutamine and L-arginine were upregulated in the AD group compared with the CN group; acetyl-L-carnitine, sphingosine 1-phosphate, palmitoylcarnitine, 5-oxoproline, uracil, uric acid, hypoxanthine, L-histidine, decanoylcarnitine, ornithine, betaine, and cortisol exhibited downregulation in the AD group compared with the CN group. The expressions of metabolites that were upregulated and downregulated are presented as boxplots in Figure 4. However, the remaining 13 components showed no significant change between the AD and CN groups, and corresponding boxplots are included in Supplementary Figure 2.

### 3.7. Discrimination and correlation analysis with plasma biomarkers

**Figure 5:**
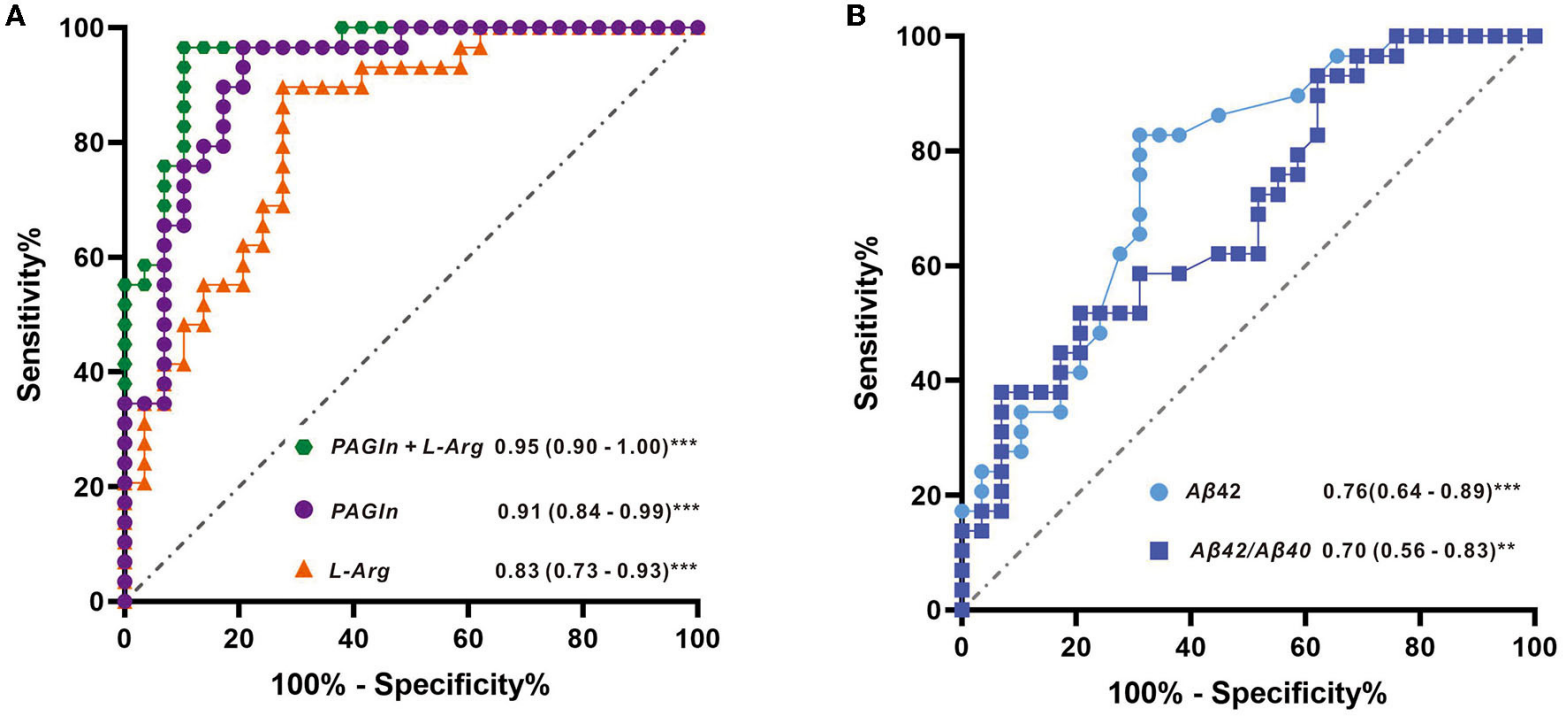
**(A)** ROC curves with metabolite features of L-arginine, PAGIn, and binary logistic regression of two metabolites (PAGIn + L-Arg) and **(B)** ROC curves with protein features of Aβ42 and the ratio of Aβ42/Aβ40.

**Figure 6:**
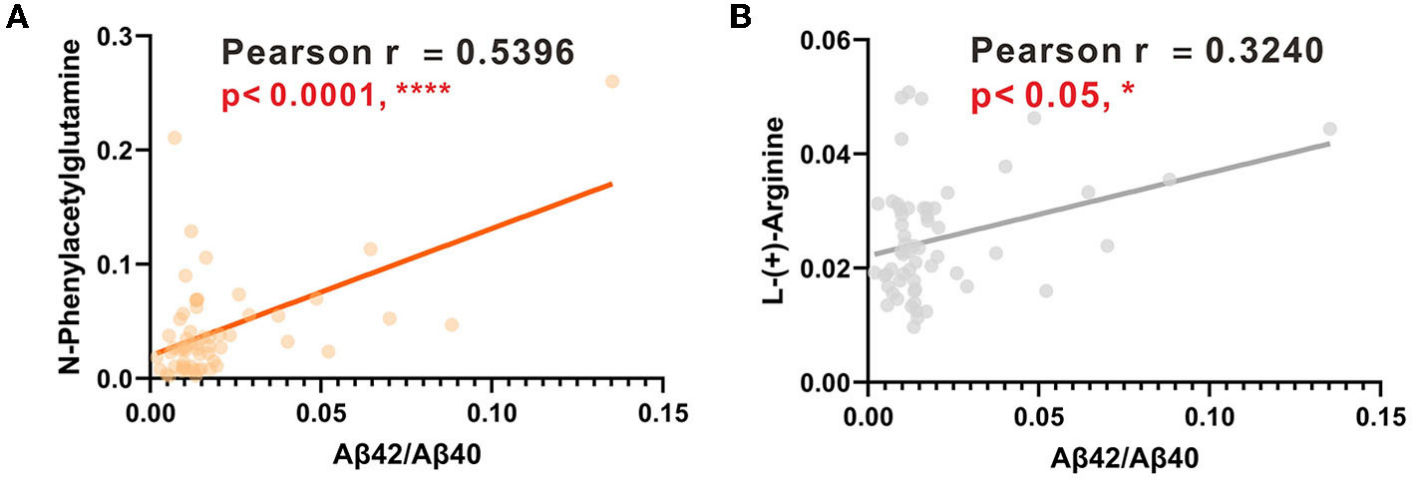
**(A)** Linear regression analysis between PAGIn and Aβ42/Aβ40. **(B)** Linear regression analysis between L-Arg and Aβ42/Aβ40.

The AUCs were utilized in this study to evaluate the diagnostic potential of various biomarkers. A value between 0 and 1 for the AUCs indicated the level of diagnostic accuracy ranging from no to great discrimination. The AUC values >0.8 were identified for phenylacetylglutamine (PAGln) and L-arginine, with AUC (PAGln) = 0.91 (95% confidence interval CI, 0.84, 0.99) and AUC (L-arginine) = 0.83 (95% confidence interval CI, 0.73, 0.93). To enhance the discrimination power of the model, a binary logistic regression was used with PAGln and L-arginine, which resulted in a model with an AUC of ROC of 0.95 (95% confidence interval CI, 0.90, 1.00). Figure 5A illustrates the ROC curves of the potential metabolic biomarkers. In addition, ROC analysis was performed to compare the discrimination ability between potential proteins and MS-discovered metabolites using Aβ42 and the ratio of Aβ42/Aβ40, respectively, with AUC (Aβ42) = 0.76 (95% confidence interval CI, 0.64, 0.89) and AUC (Aβ42/Aβ40) = 0.70 (95% confidence interval CI, 0.56, 0.83). Furthermore, the ROC curves of potential protein biomarkers are shown in Figure 5B. Pearson analysis indicated a positive correlation between Aβ42/Aβ40 and either PAGln or L-Arg. For Aβ42/Aβ40 and PAGln, *r* = 0.5396, *p* < 0.0001, while for Aβ42/Aβ40 and L-Arg, *r* = 0.3240, *p* < 0.05. Both correlation analyses indicated a statistically significant relationship between novel metabolites and the classical protein ratio (Figure 6).

## 4. Discussion

There is an unmet demand for an examination that is simple, less intrusive, and affordable in the clinical diagnosis of AD. Untargeted metabolomics presents enormous potential in the exploration of new molecules implicated in the pathogenesis of AD. Using high-resolution mass spectrometry, we found that AD was closely linked to increased plasma levels of phenylacetylglutamine (PAGIn) and L-arginine (L-Arg). Furthermore, the AD group showed lower levels of metabolites of fatty acyls, sphingolipids, and steroids, in addition to other organic acids.

PAGIn, a gut microbiota-derived metabolite, which is derived from the essential amino acid phenylalanine, has been extensively studied as a toxin in chronic kidney disease and adverse cardiovascular events (Aronov et al., 2011; Poesen et al., 2016; Nemet et al., 2020; Yu et al., 2021). Emerging evidence have demonstrated that gut microbiota dysbiosis is functionally connected to brain immune dysfunctions (Sampson and Mazmanian, 2015). Also, the increased permeability of the gut and blood–brain barrier induced by microbiota dysbiosis may mediate or affect AD pathogenesis or other neurodegenerative disorders (Jiang et al., 2017). A metabolic profiling study on Parkinson's disease (PD) using a mass spectrometry-based approach found that PAGIn was significantly elevated in the PD group as compared to the healthy controls, and its metabolic disturbances suggested that proteolytic metabolism was highly activated in PD (Shao et al., 2021). Nevertheless, limited findings have shown the dysregulation of plasma PAGIn in AD. This is our first observation documenting that plasma PAGIn is significantly elevated in AD patients, presenting an excellent discriminating power as a potential metabolite biomarker. Studies suggest that stochastic AD is associated with atherosclerosis, redox stress, inflammatory processes, and/or abnormal neurotransmitter and glucose metabolism in the brain (Yi et al., 2009). Thus, it is probable that PAGIn is involved in one or more pathophysiological processes, as previously stated. However, the underlying causes of PAGIn in Alzheimer's disease still require clarification.

L-Arg is a semi-essential amino acid that can be metabolized to form numerous bioactive molecules. Its involvement in AD is largely based on scattered information from a single pathway (Malinski, 2007). In a study examining arginine metabolism, researchers investigated three areas of the human brain: the superior frontal gyrus (SFG), the hippocampus (HPC), and the cerebellum (CE) in AD patients. They found that L-Arg was significantly elevated in the SFG area of AD patients, while L-ornithine (a product of arginase) showed a dramatic reduction in all three areas in AD (Liu et al., 2014). Interestingly, these findings were consistent with our results in the plasma metabolite investigation. The box plots illustrated that L-Arg was significantly elevated in the AD group, while its byproduct, L-ornithine, notably decreased in the AD group (Figures 4A, L). Furthermore, research has indicated that L-Arg has several direct and indirect effects on human vasculature, suggesting that it may play a crucial role in the pathogenesis of both atherosclerosis and AD. For instance, L-Arg has been found to be involved in diverse physiological and pathological processes, including neurotransmission (Chen and Chang, 2002), neurogenesis and neuroplasticity (Marcinkowska et al., 2022), cellular redox metabolism and redox stress (Perry et al., 2002; Tonnies and Trushina, 2017), inflammation (Wijnands et al., 2015), and regulation of cerebral blood flow (Matsuda et al., 2007).

In our previous study utilizing AD urine samples, an increase in potential diagnostic metabolites of uric acid, creatine, and choline was observed in the AD group as compared to the CN group. Conversely, in plasma samples, while uric acid displayed a decreasing trend, both creatine and choline indicated no significant differences between the two groups (Zhang et al., 2022). The variability in the concentration of biomarkers in body fluids is due to the kidney's crucial role in reabsorbing certain substances from urine back into the bloodstream. Recent research also demonstrated a correlation between blood biomarker concentration with urinary biomarkers (Ho et al., 2015; Kukova et al., 2019). It appears that these metabolites undergo dynamic fluctuations during certain stages of Alzheimer's disease.

Besides, different biomarkers may denote the progression of Alzheimer's disease (Teunissen et al., 2022).

In addition, we investigated the AD biomarkers of Aβ40, Aβ42, APOE4, and TAU in blood to find their association with reported results (Rachakonda et al., 2004; Niedzwiecki et al., 2020; Thijssen et al., 2022). It has been suggested that the plasma ratio of Aβ42/Aβ40 is often used to discriminate amyloid PET positive and negative individuals across the clinical AD continuum (De Meyer et al., 2020; Verberk et al., 2020). In a case–control study, researchers used the ratio of Aβ42/Aβ40 in the discrimination test and acquired a good AUC(ROC), which is above 0.8 (Thijssen et al., 2022). Based on our ELISA results, we performed the discrimination test of ROC with Aβ42 and the Aβ42/Aβ40, which resulted in AUC (Aβ42) as 0.76 and AUC (Aβ42/Aβ40) as 0.70, and this was kept in line with reported cases.

Human APOE is a glycoprotein that is highly expressed in stressed neurons, astrocytes, microglia, vascular mural cells, and choroid plexus cells. It has been suggested that low plasma levels of APOE are linked to an elevated risk of developing future Alzheimer's disease and all forms of dementia in the general population (Rasmussen et al., 2015). Also, lower plasma concentrations of APOE were supported by the finding that APOE levels are negatively correlated with Aβ levels in multiple brain regions when analyzed in non-demented individuals (Shinohara et al., 2013). In this study, AD patients held lower APOE levels as compared to the CN participants, which presented a similar trend with reported cases.

Research suggested that Tau protein lost its ability to bind to microtubules, and therefore, its normal role of keeping the well-organized cytoskeleton is no longer effective (Kolarova et al., 2012). One prospective study indicated that high plasma Tau was found in patients with AD dementia compared with cognitively normal individuals and patients with MCI (Mattsson et al., 2016). However, another study revealed a significant decrease in plasma levels of total tau among individuals with MCI compared with cognitively normal controls, with a further highly significant reduction in AD patients compared with both MCI and normal controls (Sparks et al., 2012).

In our study, we observed a decrease in plasma t-tau levels in the AD group, which differs from the trends observed in other research groups (Mattsson et al., 2016; Shen, 2020). Another study that tested the alteration of plasma tau in AD found that plasma tau partly reflects AD pathology, but there is a considerable overlap with normal aging, especially in individuals without dementia (Mattsson et al., 2016). Recent studies suggested that elevated levels of tau in the blood are not specific to AD, which could also be found in other neuron degeneration conditions, such as Parkinson's disease, frontotemporal dementia, or amyotrophic lateral sclerosis (ALS) (Neumann et al., 2006; Boeve et al., 2022; Pan et al., 2022). Thus, future studies should test longitudinal plasma tau measurements in AD.

Nevertheless, the exciting and rapid developments in plasma-based assays hold promise for prescreening in research (reducing the need for, and associated costs with, lumbar punctures and PET scans), once properly validated, which would fulfill the diagnostic purposes in clinical practice. In this study, we employed the high-resolution mass spectrometer to screen the plasma metabolites, with the optimized and stabilized LCMS method, and we were able to determine hundreds of features that enable us to perform the discovery study. We found that both PAGIn and L-Arg showed pretty good discriminate power in separating AD patients from CN individuals, which exerted their underlying possibilities in clinical diagnosis. In addition, the combination group of PAGIn and L-Arg enhanced the diagnostic power from ROC (AUC) 0.91 to ROC (AUC) 0.95, thus improving the accuracy in AD discrimination with this test.

Epidemiological studies in different populations showed an independent relationship between the development of dementia and the incidence of cardiovascular diseases (CVD), implying the presence of shared biological processes (Cortes-Canteli and Iadecola, 2020; Stakos et al., 2020). Our results indicate that both plasma PAGIn and L-arginine are significant in separating Alzheimer's disease (AD) from cognitively normal individuals. However, limited investigations suggest that PAGIn and L-arginine are associated with AD but not CVD in plasma investigation. It is interesting to observe that, using mass spectrometry in one measurement, PAGIn and L-arginine were found to be significantly elevated in AD.

Besides, we introduced the discrimination analysis with Aβ42 and Aβ42/Aβ40. The best ROC of the protein biomarker is Aβ42 with an AUC of 0.76, which was in line with the literature. Moreover, protein–metabolite interactions are of importance in cellular procedure, since these compounds could serve as co-factors for proteins to mediate protein function. This has not been investigated in our previous studies on Alzheimer's disease. It is interesting to note the positive correlations between Aβ42/Aβ40 with PAGIn or L-Arg with statistical significance. This suggests that PAGIn and L-Arg hold great promise in the diagnosis of Alzheimer's disease. These findings should be further validated in future tests using clinical specimens.

However, our study has some limitations. First, it is restricted by the lack of access to pathology reports and APOE genotype information, which impacts the scope of clinical parameters that can be included in this article. In future research, MRI could be used to visualize decreased gray matter (GM) volume in AD patients or a positron emission tomography (PET) scan could be conducted to detect amyloid deposition in AD patients, as these methods are considered the “gold standard” for assessing AD states in clinical settings, and would support the diagnosis of metabolite biomarker studies. Second, the observational nature of the study design means that the causal links between two metabolites (PAGln and L-Arg) and AD cannot be established. Instead, a functional metabolomics technique must be used to uncover the underlying pathways. Third, our study was conducted at a single location and had a biased patient selection; the sample size was also small, which means that further validation at other research centers and larger sample sizes are required. Finally, absolute quantitation analysis of differential metabolites was not performed due to a limited plasma sample, which should be done in future cohort studies to validate the findings.

## 5. Conclusion

In conclusion, our study showed that AD patients had an altered peripheral metabolism as compared to cognitively normal participants. We demonstrated the added advantage of examining metabolic expression signatures and constructing a comprehensive picture of metabolic change by examining categorization and regulatory signatures. Not only could studying additional signatures highlight potential predictive and regulatory indicators but could also uncover essential features that may have been overlooked when only investigating expression signatures. Particularly, PAGIn and L-Arg have been identified as potential essential features in metabolic alteration and showed an excellent discrimination ability in AD diagnosis. Our study also highlights a significant association between the AD protein ratio of Aβ42/Aβ40 and PAGIn or L-Arg. These findings indicate that protein biomarkers correlated with metabolites can strengthen AD diagnosis. Future studies will be required to corroborate these results and to clarify the specific roles of these metabolites in AD metabolic change.

## Data availability statement

The data presented in the study are deposited in the MetaboLights public database. This data can be found here: https://www.ebi.ac.uk/metabolights/editor/study/MTBLS8045/.

## Ethics statement

The studies involving human participants were reviewed and approved by the Ethics Committee of Shanghai Baoshan Luodian Hospital (Approval number: LDYY-KY-2020-04). The patients/participants provided their written informed consent to participate in this study.

## Author contributions

XD and QZ contributed to the conception of this article. JiY performed the experiment and wrote the manuscript. SW performed the data collection and data analysis. JuY interpreted data for the work and joined in constructive discussions. All authors have read and agreed to the published version of the manuscript.
