# Supplementary material for: Amyloid beta-correlated plasma metabolite dysregulation in Alzheimer's disease: an untargeted metabolism exploration using high-resolution mass spectrometry toward future clinical diagnosis
Source: Front Aging Neurosci. 2023 Jun 29;15:1189659. doi: 10.3389/fnagi.2023.1189659 (PMC10338932; doi:10.3389/fnagi.2023.1189659)
Supplement: Supplementary file 1 [file Data_Sheet_1.PDF]

Supplementary Material

Amyloid-beta-correlated plasma metabolites dysregulation in Alzheimer's disease: an untargeted metabolism exploration by using high-resolution mass spectrometry towards future clinical diagnosis

Jingzhi Yang #, Shuo Wu#, Jun Yang, Qun Zhang*, Xin Dong*

*** Correspondence:** Corresponding Author: dongxin@shu.edu.cn

1 Supplementary Figures

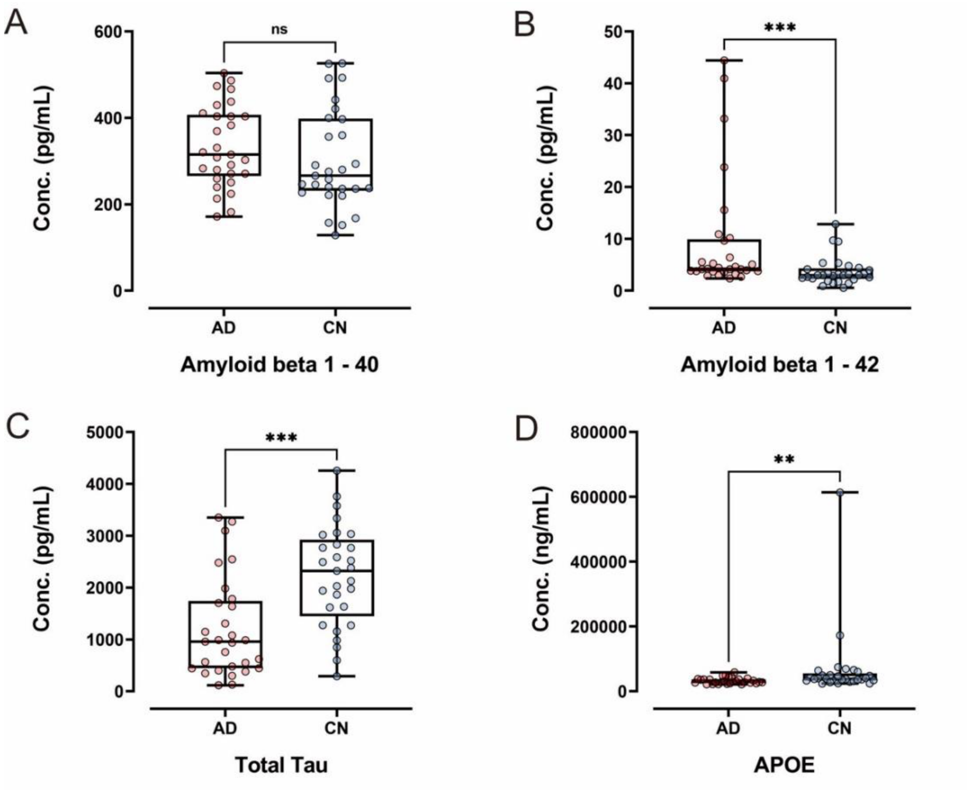

Supplementary Figure 1. Boxplots showed expression differences of (A) Amyloid beta 1-40, (B) Amyloid beta 1-42, (C) Tau, and (D) APOE. Plots showed all data points from the minimum value to the maximum value for each of group. Light pink dots represented AD patients, sky blue dots represented CN individuals, and dots were staggered and distributed on the boxes. ns: no significance, **: $p<0.05$ and ***: $p<0.001$.

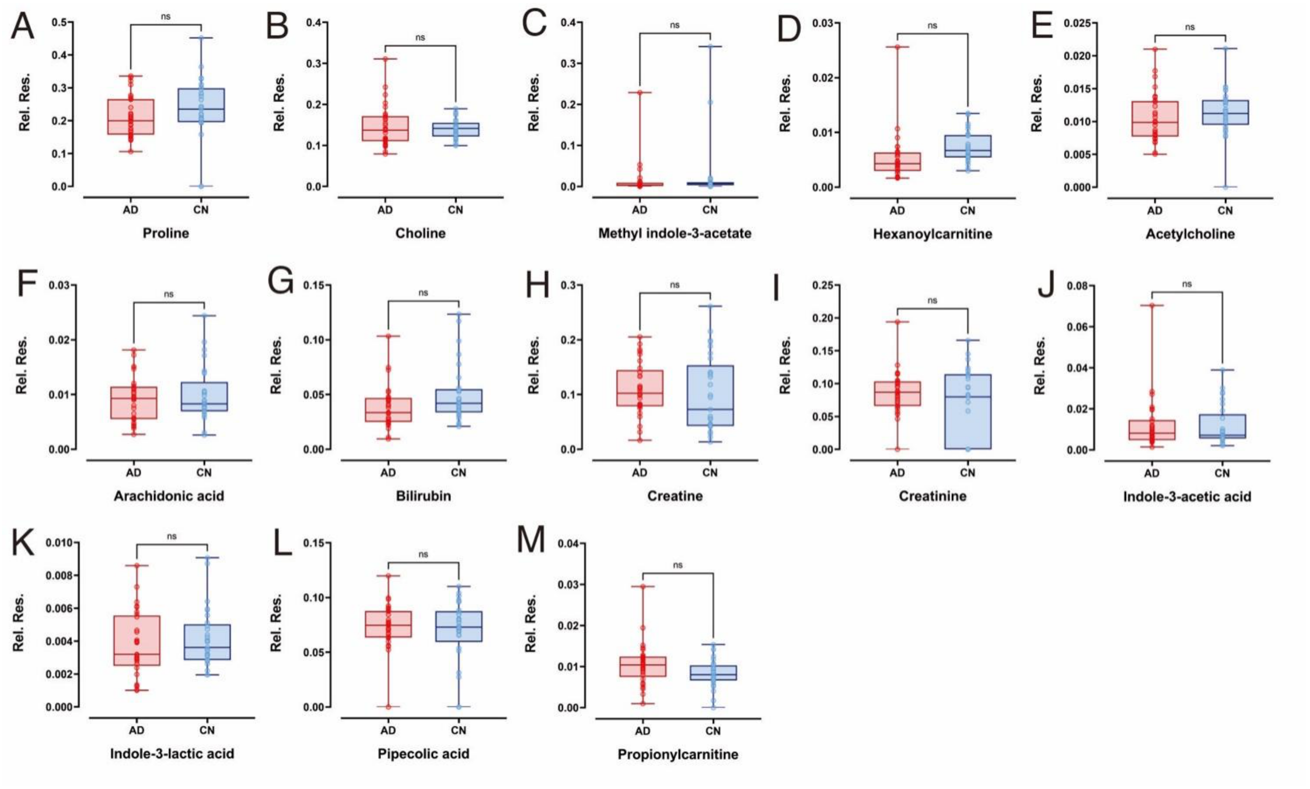

Supplementary Figure 2. Boxplots showed plasma metabolites with no significant alteration between AD group and CN group. (A) Proline, (B) Choline, (C) Methyl indole-3-acetate, (D) Hexanoylcarnitine, (E) Acetylcholine, (F) Arachidonic acid, (G) Bilirubin, (H) Creatine, (I) Creatinine, (J) Indole-3-acetic acid, (K) Indole-3-lactic acid, (L) Pipecolic acid and (M) Propionylcarnitine. Plots showed all data points from the minimum value to the maximum value for each of group. Light pink dots represented AD patients, sky blue dots represented CN individuals, and dots were vertically aligned on the boxes for each of group. ns: no significance.
